# Children’s Spontaneous Gestures Reflect Verbal Understanding of the Day/Night Cycle

**DOI:** 10.3389/fpsyg.2020.01123

**Published:** 2020-06-05

**Authors:** Caroline M. Gaudreau, Florencia K. Anggoro, Benjamin D. Jee

**Affiliations:** ^1^College of Education and Human Development, University of Delaware, Newark, DE, United States; ^2^Department of Psychology, College of the Holy Cross, Worcester, MA, United States; ^3^Department of Psychology, Worcester State University, Worcester, MA, United States

**Keywords:** gesture, astronomy, mental models, day/night cycle, embodiment

## Abstract

Understanding the day/night cycle requires integrating observations of the sky (an *Earth-based* perspective) with scientific models of the solar system (a *space-based* perspective). Yet children often fail to make the right connections and resort to non-scientific intuitions – for example, the Sun moving up and down – to explain what they observe. The present research explored whether children’s *gestures* indicate their conceptual integration of Earth- and space-based perspectives. We coded the spontaneous gestures of 85 third-grade children in U.S. public schools (*M*_age_ = 8.87 years) as they verbally explained the overall cause of the day/night cycle, the cause of sunrise, and the cause of sunset after receiving science instruction as part of a prior study. We focused on two kinds of gestures: those reflecting the Sun’s motion across the sky and those reflecting the Earth’s axial rotation. We found that participants were more likely to produce Earth rotation gestures for a topic they explained *more* accurately (the overall cause of the day/night cycle), whereas Sun motion gestures were more common for topics they explained *less* accurately (the causes of sunrise and sunset). Further, participants who produced rotation gestures tended to provide more accurate verbal explanations of the overall cause. We discuss how gestures could be used to measure – and possibly improve – children’s conceptual understanding and why sunrise and sunset may be particularly difficult topics to learn.

## Introduction

Promoting student participation and performance in STEM (science, technology, engineering, and mathematics) is an educational priority in the United States. To help more students succeed, we must better understand how students think and learn in these disciplines. In this paper, we explore how children spontaneously gesture when expressing ideas about an intensely spatial science topic, the day/night cycle. These non-verbal behaviors may reflect children’s thinking about spatial systems and, if better understood, provide a means for influencing conceptual understanding.

There is mounting evidence that spatial thinking – including mental rotation, mental transformation, and perspective taking – contributes to STEM learning outcomes (Wai et al., 2009; Lee and Bednarz, 2012; Uttal and Cohen, 2012; Heywood et al., 2013; Newcombe and Shipley, 2015; Stieff et al., 2016; Carr et al., 2017). Consider space science. To grasp fundamental ideas such as the day/night cycle, a student must connect observations from the Earth’s surface (e.g., the Sun appearing to rise in the sky) with large-scale events in our solar system (e.g., a location on the Earth becoming exposed to sunlight due to the Earth’s rotation). Mentally integrating Earth- and space-based perspectives of the solar system represents a difficult spatial thinking challenge (Heywood et al., 2013; Plummer, 2014, 2016). Besides the difficulty of mapping Earth- and space-based perspectives of the solar system, children’s own intuitions about the world (e.g., witnessing the Sun’s apparent motion) can impede their understanding (Shtulman, 2017). As children attempt to map the relations between Earth- and space-based perspectives, they may rely on inherently spatial modes of expression, such as *gesture*. Gestures often add information that is missing from verbal explanations (Goldin-Meadow, 2005, 2015; Alibali and Kita, 2010; Alibali et al., 2011, 2013; Özçalışkan et al., 2014; Waters and Beck, 2015). Highly visuospatial concepts in STEM may be more easily expressed through gesture than speech (Iverson and Goldin-Meadow, 2005; Atit et al., 2015; Stieff et al., 2016). Gestures can reduce demands on memory systems (Iverson and Goldin-Meadow, 2005) and can increase focus on a topic (Goldin-Meadow, 2015). In fact, certain concepts may depend on encoding through bodily motion and corresponding sensorimotor brain regions (Matthews-Saugstad et al., 2016; Alibali and Nathan, 2018). During learning about the day/night cycle, children can use gestures to model real or apparent motion from different frames of reference (Plummer et al., 2016).

The purpose of the present study was to explore gestures that children spontaneously produced while explaining key events in the day/night cycle. Our sample included dozens of third-grade children in U.S. public schools who were interviewed before and after completing a series of lessons about the day/night cycle as part of an earlier study (Jee and Anggoro, 2019). While the study involved three different instructional conditions, all conditions covered the same concepts and involved instruction with a 3D model of the Earth–Sun system (see Jee and Anggoro, 2019, for further detail).

The current analyses focused on participants’ responses to questions about the cause of (1) the overall change from day to night, (2) sunrise, and (3) sunset. If a participant grasped the scientific explanations from the lessons, then they should provide the same causal explanation for all three topics – namely, the Earth’s eastward rotation as seen from a space-based perspective. However, if a participant is focused on the Sun’s apparent motion from an Earth-based perspective, or confused about whether the Sun moves, they may provide inadequate or incomplete verbal explanations. Such confusions and omissions may be more frequent for sunrise and sunset, which invoke an Earth-based frame of reference and are labeled in terms of the Sun motion, than for the overall day/night cycle.

We coded participants’ non-verbal behavior as they responded to relevant interview questions, looking specifically for the occurrence of two kinds of gestures: (1) *Earth rotation gestures* that represent the rotating motion of the Earth from a large-scale, space-based perspective and (2) *Sun motion gestures* that indicate movement of the Sun across the sky. We considered two main ways in which gestures could relate to verbal understanding:

1. Gesture as a *mirror* that reflects *existing*, verbalized knowledge (e.g., Alibali, 2005; Plummer et al., 2016).
2. Gesture as a *window* into ideas that are not yet (or cannot be) expressed in speech. In this sense, gestures indicate burgeoning conceptual change, predicting *future* breakthroughs in verbalized knowledge (e.g., Church and Goldin-Meadow, 1986; Goldin-Meadow, 2003).

If gesture acts like a *mirror*, then Earth rotation gestures should be especially frequent when participants verbalize high levels of causal understanding, and Sun motion gestures should be more frequent when verbalized understanding is low. A finer-grained prediction is that children who make Earth rotation gestures should explain the day/night cycle more accurately than should children who do not, both at pretest and at posttest. If, however, gestures are a *window* into emerging knowledge, then Earth rotation gestures at pretest should predict verbalized understanding following instruction, at the posttest.

## Materials and Methods

### Participants

Participants included 85 third-grade U.S. public school students (*M*_age_ = 8.87, *SD* = 0.50, 57% female) who completed a series of lessons about the day/night cycle as part of a larger study (Jee and Anggoro, 2019) between October 2015 and December 2016. The demographic distribution of this sample reflected that of the school district from which the sample was acquired, 14.9% were African American, 7.5% Asian, 40.8% Hispanic, 0.2% Native American, 32.5% White, and 4.1% multiracial, non-Hispanic.

### Interviews and Coding Procedure

All participants completed pretest and posttest interviews about the day/night cycle. Each interview lasted about 15–20 min per child and was videotaped by a trained research assistant. Interview questions were administered verbally by the research assistant. The questions asked participants to explain the cause of (1) the overall change from daytime to nighttime, (2) sunrise, and (3) sunset (see Jee and Anggoro, 2019, for further details). Table 1 provides the relevant interview questions along with the two knowledge components that were used to score participants’ verbal responses for each topic. Children scored 1 point for each knowledge component they correctly verbalized. Intercoder reliability for verbal knowledge scoring was established through independent coding trials, followed by reliability analyses, discussion, and refinement of the coding criteria. All coders obtained reliability of 0.80 or higher (Krippendorff’s *α*) with the other coders on two consecutive rounds of four to six interviews. Reliability ranged from 0.82 to 0.96 across all knowledge components (Jee and Anggoro, 2019).

**TABLE 1:** Interview questions and knowledge components for scoring responses.

Topic			
	Overall cause	Sunrise	Sunset
Question(s)	What causes the change from daytime to nighttime?	Every day in [a large city in Northeastern United States], an event happens that we call “sunrise.” It looks like this [show video of sunrise]. Have you seen sunrise before? What is sunrise? Why does sunrise happen? Why does sunrise happen in the east?	Every day in [a large city in Northeastern United States], an event happens that we call “sunset.” It looks like this [show video of sunset]. Have you seen sunset before? What is sunset? Why does sunset happen? What makes sunset happen in the west?
Knowledge components	The Earth spins/turns/rotates Rotation causes places to face the Sun at different times.	The Earth rotates east/a place rotates toward the Sun. The Earth rotates until a place begins to face the Sun.	The Earth rotates east/a place rotates away from the Sun. The Earth rotates until a place begins to face away from the Sun.

**FIGURE 1:**
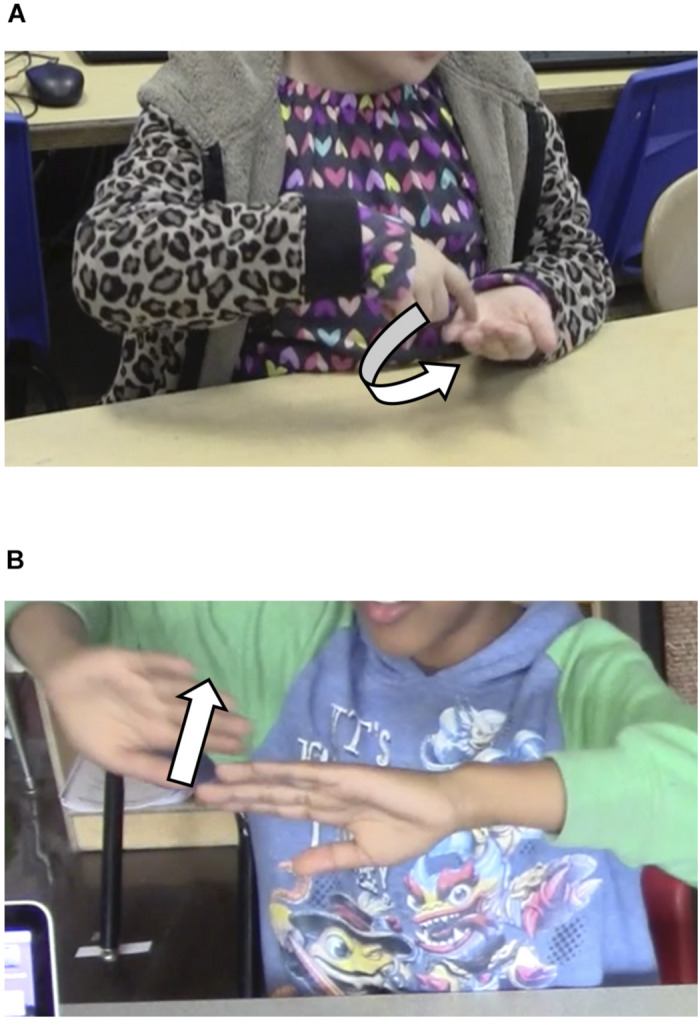
Examples of **(A)** Earth rotation and **(B)** Sun motion gestures. Arrow indicate movement of finger or hand.

Children’s gestures during the interviews were also coded. Rules for coding gestures were made stringent: (a) a complete gesture necessitated a clear break from any fidgeting of the hand or fingers before or after the gesture, and (b) any gestures made toward technology or items in the classroom were not included, on the basis that not all children had the same resources available during the interviews. Gestures were categorized as one of two types (see Figure 1), based largely on work by Goldin-Meadow (2005) and Plummer (2014). *Earth rotation* gestures involved children using the finger, hand, or arm to represent the rotating motion of the Earth. For the purpose of this study, only instances where children explicitly mentioned “the Earth” in their verbal responses were considered when coding rotation gestures, to avoid ambiguity concerning what children’s gestures were intended to represent. In *Sun movement* gestures, children used the finger, hand, or arm to indicate leftward, rightward, upward, or downward movement of celestial objects. In nearly all instances observed, these gestures referenced the Sun motion (i.e., children mentioned “the Sun” in their verbal response). Coders made notes during coding, so only movement gestures that included an explicit verbal reference to the Sun were analyzed for the current study. The coders established interrater reliability (Krippendorff’s α = 0.83) using 10% of the 170 pretest and posttest interviews.

## Results

### Verbal Explanations

Participants’ knowledge of the three topics was scored in terms of the number of components they included in their verbal explanations (see Table 2). We conducted a 2 (Session: pre, post) × 3 (Topic: overall cause, sunrise, and sunset) repeated-measures ANOVA to compare children’s knowledge of the overall cause of the day/night cycle, sunrise, and sunset at pretest and posttest. Assumptions for Mauchly’s *W* were not met, so results from the Huynh–Feldt model are reported, as suggested by Howell (2002) and Field (2013). Results revealed main effects of Session, *F*(1, 84) = 25.48, *p* < 0.001 (participants had higher knowledge at posttest than pretest), and Topic, *F*(1.45, 121.48) = 177.71, *p* < 0.001 [participants had higher knowledge for the overall cause than for sunrise (*p* < 0.001) and sunset (*p* < 0.001) but did not differ in their knowledge of sunrise and sunset (*p* = 0.697)]. There was also a significant interaction between Session and Topic, *F*(1.58, 132.73) = 5.24, *p* = 0.011 – although knowledge of each topic increased significantly, *F*s > 13.0, *p*s < 0.001, the increase was greater for the overall cause of the day/night cycle than for sunrise or sunset.

**TABLE 2:** Descriptive statistics for children’s knowledge and gestures by topic.

Variable	Topic		
	Overall cause	Sunrise	Sunset
**Knowledge components**			
Pretest	0.72 (0.77)	0.05 (0.26)	0.05 (0.21)
Posttest	1.35 (0.70)	0.41 (0.64)	0.39 (0.62)
**Earth rotation gesture**			
Pretest	32 (37.6%)	15 (17.6%)	17 (20.0%)
Posttest	32 (37.6%)	18 (21.2%)	14 (16.5%)
**Sun motion gesture**			
Pretest	19 (22.4%)	37 (43.5%)	41 (48.2%)
Posttest	6 (7.1%)	29 (34.1%)	23 (27.1%)

*N = 85. Number of children (and percentage) who made a gesture reported for each type of gesture.*

### Relations Between Verbal Explanations and Gestures

Gestures were coded categorically; children received a 1 if they made a given type of gesture when verbally explaining a specific topic and a 0 if they did not make the gesture. Table 2 shows the number of children who made a given type of gesture for each topic at pretest and posttest. Earth rotation gestures appear to be more common during explanations of the overall cause than for sunrise and sunset. Sun motion gestures, however, were more likely during explanations of sunrise and sunset. This pattern for Sun motion gestures appears to persist from pretest to posttest despite an overall decrease in the frequency of Sun motion gestures.

We planned to test how gestures related to verbally expressed knowledge by comparing the average knowledge of participants who did vs. did not produce a given type of gesture for each topic. However, gesture frequency was less than 25% in some cases (e.g., only 17.6% of participants made a rotation gesture for the sunrise topic at pretest). To avoid comparing wildly uneven group sizes and the consequent loss of statistical power (e.g., Rusticus and Lovato, 2014), we divided participants into Earth rotation gesturers and non-gesturers on the basis of their gestures for the overall cause topic – the topic for which rotation gestures were most frequent^1^. We then tested whether participants who made an Earth rotation gesture for the overall cause topic differed in their verbally expressed knowledge for each of the three topics. We applied a similar rationale to divide participants into Sun motion gesturers and non-gesturers. Specifically, we tested whether participants who made a Sun movement gesture while describing sunrise or sunset – topics for which Sun motion gestures were most frequent – differed in their verbally expressed knowledge for each of the three topics. Table 3 shows the mean verbal knowledge scores on each topic for the Earth rotation and Sun motion gesture groupings.

**TABLE 3:** Mean verbal knowledge of each topic for gesture and non-gesture groups at pretest and posttest.

Gesture grouping	Topic		
	Overall cause	Sunrise	Sunset
**Earth rotation gesture for overall cause**	**Pretest**		
Made gesture, *n* = 32	0.94 (0.80)	0.09 (0.39)	0.00 (0.00)
Did not make gesture, *n* = 53	0.58 (0.72)	0.02 (0.14)	0.08 (0.27)
	**Posttest**		
Made gesture, *n* = 32	1.53 (0.51)	0.47 (0.72)	0.34 (0.48)
Did not make gesture, *n* = 53	1.25 (0.78)	0.38 (0.60)	0.42 (0.69)
**Sun motion gesture for sunrise**	**Pretest**		
Made gesture, *n* = 37	0.81 (0.70)	0.03 (0.16)	0.03 (0.16)
Did not make gesture, *n* = 48	0.65 (0.81)	0.06 (0.32)	0.06 (0.24)
	**Posttest**		
Made gesture, *n* = 29	1.28 (0.70)	0.41 (0.68)	0.38 (0.62)
Did not make gesture, *n* = 56	1.39 (0.70)	0.41 (0.63)	0.39 (0.62)
**Sun motion gesture for sunset**	**Pretest**		
Made gesture, *n* = 41	0.85 (0.73)	0.05 (0.22)	0.05 (0.22)
Did not make gesture, *n* = 44	0.59 (0.79)	0.05 (0.30)	0.05 (0.21)
	**Posttest**		
Made gesture, *n* = 23	1.43 (0.74)	0.52 (0.73)	0.39 (0.58)
Did not make gesture, *n* = 62	1.32 (0.74)	0.37 (0.61)	0.39 (0.64)

*N = 85. Standard deviations in parentheses.*

### Earth Rotation Gestures

At pretest, participants who made an Earth rotation gesture for the overall cause topic tended to have higher knowledge of the overall cause than did participants who did not make the rotation gesture, *t*(83) = 2.10, *p* = 0.039. However, these rotation gesturers had about the same level of knowledge of sunrise as participants who did not make the rotation gesture, *t*(83) = −1.28, *p* = 0.205. Verbal knowledge of sunset was very low overall at pretest. Rotation gesturers expressed no knowledge of sunset, whereas non-rotation gesturers had slightly higher verbal knowledge, *t*(52) = 1.60, *p* = 0.044 (adjusting degrees of freedom in light of unequal variances in Levene’s test, *F* = 12.10, *p* = 0.001).

At posttest, participants who made an Earth rotation gesture for the overall cause topic tended to have higher knowledge of the overall cause than did those who did not make the rotation gesture, *t*(82.52) = −2.04, *p* = 0.044 (adjusting degrees of freedom in light of unequal variances in Levene’s test, *F* = 8.04, *p* = 0.006). These rotation gesturers had about the same level of knowledge of sunrise as participants who did not make the rotation gesture, *t*(83) = −0.91, *p* = 0.528, and likewise for sunset, *t*(83) = 0.513, *p* = 0.610.

### Sun Motion Gestures

At pretest, participants who made the Sun motion gesture when explaining sunrise had about the same knowledge level of the overall cause topic as participants who did not make the Sun motion gesture, *t*(83) = −0.98, *p* = 0.328. These Sun motion gesturers also expressed about the same level of knowledge of sunrise as the non-gesturers, *t*(83) = −0.614, *p* = 0.541, and likewise for sunset, *t*(83) = 0.759, *p* = 0.450.

At posttest, participants who made a Sun motion gesture for the sunrise topic had about the same knowledge level of the overall cause as participants who did not make the Sun motion gesture, *t*(83) = 0.726, *p* = 0.470. These Sun motion gesturers also expressed about the same level of knowledge of sunrise as the non-gesturers, *t*(83) = −0.021, *p* = 0.983, and likewise for sunset, *t*(83) = 0.095, *p* = 0.924.

We obtained the same pattern of results when we sorted participants into Sun motion gesture and non-gesture groups on the basis of whether they made the Sun motion gesture when explaining *sunset* (*p*s > 0.114, see Table 3 for mean knowledge components for gesturers and non-gesturers).

### Do Pretest Rotation Gestures Predict Posttest Understanding?

To test whether gestures serve as a *window* into children’s understanding, we tested whether pretest gestures predicted verbal knowledge at posttest. We focused on Earth rotation gestures, which signify the underlying cause of the day/night cycle. We conducted an ANCOVA with children’s Earth rotation gestures predicting their posttest knowledge of the overall cause concept, controlling for children’s knowledge of the overall cause concept at pretest. The analysis revealed that Earth rotation gestures at pretest *did not* predict verbal knowledge at posttest, *F*(1, 84) = 0.365, *p* = 0.547.

## Discussion

The current study examined whether and how children’s spontaneous use of gesture relates to their explanations of the day/night cycle. Overall, participants used more Earth rotation gestures when explaining the overall cause of the day/night cycle – a topic for which both initial and eventual knowledge was relatively high. In fact, participants who made Earth rotation gestures tended to express *greater knowledge* than those who did not. Participants made more Sun motion gestures when explaining sunrise (though not for sunset, discussed further below) – topics for which initial and eventual knowledge was relatively low. Yet participants who made Sun motion gestures did not differ in verbally expressed knowledge from those who did not. As a whole, we did not observe a mismatch between participants’ speech and gesture. Thus, in the present case, gestures generally *mirrored* the knowledge that children expressed verbally, rather than serving as a window into future knowledge gains.

By the posttest, the participants had been taught repeatedly about one essential fact: the Earth’s rotation causes the change from daytime to nighttime. Nevertheless, participants were more likely to correctly explain the overall cause of the day/night cycle if they simultaneously expressed the Earth rotation gesture than if they did not. This finding supports the idea that children may require some physical bodily motion to fully encode abstract concepts – enactment theory (Matthews-Saugstad et al., 2016). The Earth’s rotation, which can only be witnessed from outer space, may be better conceptualized with the enactment of a corresponding gesture. Gesture is distinct from other bodily movements in its unique ability to combine both physical and abstract elements (Goldin-Meadow, 2003, 2006). Whereas some scientific processes can be physically experienced with the body, other science concepts have abstract elements that cannot be experienced this way. For example, while a child may be able to experience gravity by jumping off the top of a box, it is impossible to physically experience the Earth’s rotation. Gesture allows children to use their bodies to simulate such events (Goldin-Meadow, 2003, 2006; Novack and Goldin-Meadow, 2015) and could help them verbalize their understanding of spatial elements and relationships.

The participants also received repeated instruction about how the Earth’s rotation causes sunrise and sunset. However, the posttest knowledge results show that participants tended to have less knowledge of these topics than for the overall cause of the day/night cycle. We suspect that participants struggled to connect their everyday experiences of witnessing an apparent Sun motion (i.e., the Sun going “up” in the morning) with the space-based perspective that the Sun is actually stationary, in part because of the misleading terms sun*rise* and sun*set.*

Interestingly, there was no relationship between participants’ Sun motion gestures and their verbal-explanatory accuracy for either sunrise or sunset. Thus, whereas Earth rotation gestures signified understanding of the day/night cycle, Sun motion gestures did not clearly signify confusion about the cause of sunrise or sunset. This asymmetry may be due to the fact that the Sun motion gesture could reflect that a child is merely considering the Sun’s apparent motion from an Earth-based perspective *or* that they actually believe that the Sun moves up and down – an intuitive but causally incorrect idea. In either case, the expression of the Sun motion gesture suggests that the child is focused on an Earth-based perspective of the day/night cycle, perhaps at the cost of forming a robust space-based representation that relates the Earth rotation to sunrise and sunset.

The current study has some important limitations. Firstly, children were never instructed to gesture in any way during the interviews. Because there was no manipulation of children’s gestures, we cannot draw conclusions about causal links between gesture and understanding. An intriguing possibility is that encouraging Earth rotation gestures could enhance a student’s understanding and their subsequent verbal explanations of the day/night cycle (see also Plummer, 2014). Indeed, people who are instructed to gesture when solving a spatially intense science problem, such as building a geologic block diagram, performed better on a subsequent spatial reasoning task than people who were prohibited from gesturing (Atit et al., 2015). Further research is needed to test this possibility in young children’s astronomy learning.

Another limitation of the study is that there were small, but potentially important differences in the phrasing of certain interview questions. For the sunrise concept, children were asked, “Why does sunrise happen?” but for sunset, “What makes sunset happen?” The “why” phrasing for the sunrise question could have invited Earth-based and teleological responses such as “Sunrise happens so we wake up and go to school” or “Sunrise happens so a new day can start.” The question about sunset may have invited more causal-mechanistic thinking, as the Earth’s rotation is what *makes* sunset happen. So a small difference in wording could have affected how participants thought about the events in question. Future research should consider this issue when designing tests to explore the effect of children’s gestures on their knowledge of science concepts.

Finally, while the current results suggest gesture was a *mirror*, participants’ actions during other parts of the interview could have revealed a window into knowledge. For example, children’s modeling of the Earth and Sun motion using 3D balls could have served as a window into conceptual understanding. Future research could explore other components of the interview to capture a more complete picture.

Using gestures does not require expensive technology or extensive teacher training. Simply promoting the use of gesture in classrooms could be effective in improving children’s understanding of highly spatial, abstract scientific concepts. Teachers can remind children to “use their hands” or demonstrate the kinds of gestures that students should use while learning concepts in class (e.g., Stieff et al., 2016). For example, when teaching about the day/night cycle, teachers might ask children to make an Earth rotation gesture with their hands while explaining to a peer how the Earth moves. Although we cannot say whether gesture *causes* deeper space science knowledge, the positive association between gesture and verbal knowledge suggests that gestures could be a contributing factor.

Beyond space science, gesture can support children’s learning in other STEM domains. The geosciences (e.g., Atit et al., 2015) and organic chemistry (Stieff et al., 2016) offer a number of rich opportunities for incorporating gesture during instruction. Children also benefit from gestures during math lessons. Cook et al. (2008) found that children learned a mathematical concept better when they gestured during instruction rather than only speaking. Gesture may also be applied to other highly spatial, abstract domains, such as geometry, engineering, and architecture. Further research into direct applications of gesture for different scientific concepts will help inform recommendations for educators.

Gesture may serve an important role in the encoding of scientific information. In the current study, third graders who made Earth rotation gestures were more likely to verbally explain the day/night cycle than were those who did not produce this gesture. Gesturing to express highly spatial topics, such as the day/night cycle, may support children’s knowledge acquisition. With additional research, gesture could be harnessed as a tool for instruction – a way to help people encode and express STEM concepts at a deeper level.

## Data Availability Statement

The datasets generated and analyzed for this study can be found in FigShare: https://doi.org/10.6084/m9.figshare.11592060.v1.

## Ethics Statement

The studies involving human participants were reviewed and approved by the Committee on Human Subjects (IRB), College of the Holy Cross. Written informed consent to participate in this study was provided by the participants’ legal guardian/next of kin.

## Author Contributions

CG and BJ conducted the statistical analyses. CG wrote the first draft of the manuscript. FA and BJ provided the critical revisions. CG and research assistants coded the video data. All authors conceptualized the idea for the project, contributed to the development and refinement of the gesture coding scheme, interpreted findings and outlined the manuscript, and approved the final version of the manuscript for submission.

## Conflict of Interest

The authors declare that the research was conducted in the absence of any commercial or financial relationships that could be construed as a potential conflict of interest.

## Footnotes

1 This sorting method does not distinguish participants who made no gesture and participants who made Sun motion gestures – both are assigned to the non-rotation-gesture group. We chose this method to preserve statistical power. Few participants made Sun motion gestures, and these few had similar verbal knowledge scores to the non-gesturers.
